# The Learned Ass

**Published:** 1896-02

**Authors:** 


THE LEARNED ASS.—At a meeting of the Chicago Medico-Legal Society recently (January 11th, ult.), a number of papers on crime and criminals were read; among them, one by the editor of the TEXAS MEDICAL JOURNAL, in which a plea was made for reform in criminal jurisprudence. This plea was based upon facts and figures, which show that the present system, either through inherent defect or lack of enforcement, is inefficient, and neither affords protection to society, nor diminishes the number of criminals, but on the contrary, crime is alarmingly on the increase; that it is the feeling of insecurity thereby engendered that leads to the occasional lynchings. It was urged that instead of the indiscriminate meting out of punishment alike to all criminals of a class, without reference to their condition, age, sex or environments, criminals should be classified, and each treated or dealt with, with reference to his cure or reformation and reclamation to a life of usefulness. The born criminal, the irreclaimable sinner to be regarded and treated as a permanent enemy to society and imprisoned for life; that, as under existing laws all other rights are forfeited,—that of procreation should be taken away. In other words, that he be asexualized, not as a punishment, but as protection to future generations against hereditary transmission of criminal propensity. The twelve hundred criminal descendants of the well known Jukes family being cited as an illustration of what might be prevented by timely asexualization.

In the discussion which followed, a Mr. W. S. Elliott, a lawyer of Chicago, amongst other things—some equally as absurd—said "Castration as a punishment for crime would be considered, without question, an unusually harsh and cruel punishment; it is inhibited by the Constitution of the United States, and so far as I know, by that of every State in the Union. It would be unconstitutional, therefore, illegal, and it is absolutely impossible to make it constitutionally legal by legislative enactment by any legislature of any enlightened State. That they might be able to do it in Texas I deem possible, perhaps, but in Illinois, or the great States where intelligence is moving forward, a men would never consent to such wicked and outrageous proposition as castration as a punishment for crime."

That is to say—he believed it would be possible in *Texas* or in any *other un*enlightened (?) State to make anything, however unconstitutional and illegal, "constitutionally legal" by legislative enactment, although it is "absolutely impossible" to do so, in an enlightened State!

Here's legal logic for you! In the attempt to snarl at Texas this prig showed something besides his teeth—the ears, for instance—and committed an unpardonable solecism. In other words, he made an ass of himself. But the young man grew eloquent further along. He said:

"I do not think that any of us have yet arrived at that degree of perfection where we dare reach out our hand and lay it upon any single fellow being and say, you stand where we may deprive you of that which God invested you with first of all, when he said, 'Increase, multiply and replenish the earth.' If you have that right, you have right to kill a man, and you have the same right to kill him that you have to castrate him—no greater and no less in one instance than in the other."

Is not the State every day exercising the "right," whether with Mr. Elliott's consent or not—to "kill a man?" That is just what is objected to, and a method more humane and less questionable is proposed as a substitute.

When it is borne in mind that it was not proposed to castrate any but the murderer and rapist *instead of hanging or burning them*, the force, beauty, wisdom and application of the young man's remarks become impressive. To multiply is just what this class must not be permitted to do, and "replenishing the earth,"—with their sort—like the Jukes—is just what we don't want them to do, and to prevent which is the end and object of the recommendation.

This limb of the law we hope is not a representative specimen of the Illinois "intelligence," if so he was prematurely turned loose—plucked green.

